# Accompaniment on the edge: What can the US learn from Latin America about contested abortion care?

**DOI:** 10.1371/journal.pgph.0001922

**Published:** 2023-05-22

**Authors:** Alhelí Calderón-Villarreal, Bianka Itzel Verduzco Carrasco, Joseph Friedman, Raffaela Schiavon

**Affiliations:** 1 Department of Family and Preventive Medicine, University of California, San Diego (UCSD), San Diego, California, United States of America; 2 School of Public Health, San Diego State University (SDSU), San Diego, California, United States of America; 3 Facultad de Medicina, Universidad Autónoma de Coahuila (UAdeC), Torreon, Mexico; 4 Colectiva Feminista de Malas Estudiantes, Tijuana, Mexico; 5 Facultad de Humanidades y Ciencias Sociales, Universidad Autónoma de Baja California (UABC), Tijuana, Mexico; 6 Facultad de Filosofía y Letras, Universidad Autónoma de Sinaloa (UAS), Tijuana, Mexico; 7 University of California, Los Angeles (UCLA), Los Angeles, California, United States of America; 8 Former Director of Ipas-Mexico, Mexico City, Mexico; 9 Independent Consultant in Sexual and Reproductive Health, Mexico City, Mexico; PLOS: Public Library of Science, UNITED STATES

## Abstract

The US has stood as a reference point for abortion rights in the Americas since 1973, however in 2022, the US Supreme Court revoked the constitutional right to abortion. Facing similar circumstances, a huge number of grass roots accompanist networks have arisen throughout Latin America. These collectives are typically organized loosely within state and national networks that provide training and medication/supplies and promote advocacy and the expansion of new collectives. Extensive evidence and lived experience support the safety and effectiveness of self-managed medication abortion. Much can be learned from the Latin American accompanist model in the modern struggle for reproductive justice in the US. Accompaniment networks in Mexico have provided transborder abortion services—via misoprostol delivery—to US-based women living in states that required long travel or high costs to access services. Now, these transborder services will take on a new level of significance. Guaranteeing safe and low-cost access to abortion services is a key tenet of reproductive justice. Instead of relying on the political process alone to eventually provide abortion access through legal channels, an accompanist model provides an icon of resistance to oppressive legal shifts, and directly provides services to women.

Since the *Roe vs*. *Wade* ruling in 1973, the United States (US) has stood as a reference point for abortion rights in the Americas. However, after 50 years—and during a massive wave of abortion advocacy in the region—the US Supreme Court revoked the constitutional right to abortion. This affects the largest national population of women of reproductive age in the Americas (>80 million), stripping away rights in about half of US states.

During this half-century, women in Latin American countries have faced some of the most punitive abortion laws seen globally, and in response, have evolved a set of nuanced and diverse strategies to assert reproductive rights in a contested and ambiguous medico-legal landscape. Now—in the logistical and legislative chaos of the post-*Roe* era—the US is facing a set of similar challenges. Therefore, there is much that US healthcare providers, activists, and legislators can learn from the experience of Latin America.

## Accessing abortion across borders

Latin America features a diverse set of legislative approaches to abortion services, varying within and between countries. Abortion remains heavily criminalized in most of the region, with some exceptions. Cuba and Guyana legalized abortion access in 1965 and 1995, respectively. Abortion services have been legalized in Mexico City since 2007, followed by legalization or decriminalization in Uruguay (2012), nine Mexican States–Oaxaca, Hidalgo, Veracruz, Baja California, Colima, Sinaloa, Guerrero, Baja California Sur, and Quintana Roo (2019–2022)–Argentina (2020), and Colombia (2022). In Mexico, the Supreme Court declared the criminalization of abortion unconstitutional at the national level in 2021.

These differences have created a dynamic within which accessing abortion services requires crossing national or state-level borders for many women [1], similar to the post-*Roe* landscape of the US. Such situations created inequalities in access to abortion along lines of socioeconomic status, ethnicity, and other aspects of social advantage [2, 3].

When first-trimester abortion was legalized in Mexico City, approximately 7% of the country’s population suddenly had local, legal access, which was also made available free-of-charge to women from other states or countries. However, utilization from afar has been low. Even among women living close to the border with Mexico City—who could theoretically use public transportation to reach free abortion services—access disparities are profound, with each additional 15-minutes of required travel time associated with a 1/3 reduction in effective access to abortion care [2]. There is also a sharp socioeconomic gradient, wherein those women who are able to travel from outside of Mexico City to utilize abortion care are much more highly educated, and from wealthier neighborhoods, relative to their counterparts who are unable to access care [2].

Similar trends have been noted throughout the Americas; where abortion is criminalized, it is *de facto* only criminalized for the most vulnerable women, such as members of low-income or minoritized racial/ethnic groups. The Latin American experience has shown that, as in the US, women with more resources will likely continue to access abortion services despite criminalization, via travel, or by accessing private abortion services [1, 2, 4].

## The critical role of grass roots accompaniment

In this context, grass roots accompaniment has emerged as a key strategy of resistance and reproductive justice. This approach centers around the low-barrier provision of information regarding medication abortion and direct accompaniment during the process. It sometimes also includes the provision of medication for abortion outside clinical settings, with the guidance of a network of volunteer community-based activist ‘accompanists’ (*acompañante* or *socorrista*). Accompanists are typically feminist women, trans men, or gender-neutral people, who receive training within the accompanist community [5]. The accompanist provides misoprostol (available over-the-counter in much of Latin America), misoprostol/mifepristone or instructions on how to obtain them, gives detailed information about how they should be taken to induce abortion, and follows along with the accompanied person—either virtually or in-person—as she undergoes the process. Of note, even where abortion itself is criminalized, providing information and pre-post abortion services are usually legal [6]. Relationships are often maintained with physicians in the community who are willing to respond to emergencies, or provide follow-up care, although often clinicians do not participate directly. These collectives are typically organized loosely within state and national networks that provide trainings and promote advocacy, emphasizing the guiding principles of women´s autonomy, horizontality, dignity and safety [7].

WHO guidelines support self-managed medication abortion in the first trimester without the direct supervision of a health-care provider–when practiced with accurate information, quality medications and access to health services when needed or desired [8]. Ample published evidence and extensive lived experience supports the safety and effectiveness of self-managed medication abortion [4, 9–11] and accompaniment group support outside of clinical settings [12–14].

The avoidance of legal penalties is a core element of accompanist guidance. Accompanied people are instructed to take misoprostol buccally or sublingually to avoid subsequent detection, as vaginal administration can leave discoverable pill fragments, and advised that any observable symptoms are indistinguishable from a spontaneous miscarriage, which are commonplace. These strategies allow follow-up healthcare to be sought, minimizing risk of prosecution. Nevertheless, it is important to note that the criminalization of pregnant people who experience miscarriage does occur, and has been increasing.

Accompaniment networks in Latin America are autonomous and have varying internal policies, for instance, whether or not health professionals participate, if interaction with accompanied persons occurs in-person or digitally, the degree of political activism vs. service provision, and which specific services (e.g., transportation, medications) are provided [14].

## Building a resistance to the loss of reproductive rights in the US

Recent years have witnessed a profound increase in activism around reproductive rights in Latin America, called *La Marea Verde* (the green wave) for the green handkerchief symbol used widely by pro-choice activists. The ‘accompanist’ figure—disseminated massively by social media—has served as an icon in this movement, in addition to providing information and services for women who would otherwise have few options for safe abortion.

Lessons drawn from the Latin American experience can be tailored to distinct US contexts. For instance, intense state surveillance and criminalization of women’s reproductive choices in Texas and Louisiana mirror long-standing practices in Honduras and Nicaragua. Access to misoprostol in the US requires a prescription or interfacing with the black market, which can limit access and inflate medication prices. Mail-based and transborder services may therefore play a critical role. Notably, legal abortion has been virtually eliminated in several US states proximate to the border with Mexico, where misoprostol remains cheap and readily accessible. The potential for transborder accompanist networks has already been established by pioneering groups in Mexico providing services to US-based women in states requiring long travel or high costs to access services [15]. Moving forward, these networks may serve as a key source of knowledge and practical experience in accessing care in contested legal landscapes.

Guaranteeing safe and low-cost access to abortion services is a human rights issue, a key tenet of reproductive justice, and a critical matter for health equity. Instead of relying on the political process alone to eventually provide abortion access through legal channels, the Latin American experience has demonstrated that an accompanist model provides an icon of resistance to oppressive legal shifts, and directly benefits to women who would otherwise have no safe options for accessing abortion-related healthcare.
